# SARS-CoV-2 RNA Detection in Wastewater and Its Effective Correlation with Clinical Data during the Outbreak of COVID-19 in Salamanca

**DOI:** 10.3390/ijms25158071

**Published:** 2024-07-24

**Authors:** Ángel Emilio Martínez de Alba, María Eugenia Morán-Diez, Juan Carlos García-Prieto, Juan García-Bernalt Diego, Pedro Fernández-Soto, Esteban Serrano León, Víctor Monsalvo, Marta Casao, María Belén Rubio, Rosa Hermosa, Antonio Muro, Manuel García-Roig, Enrique Monte

**Affiliations:** 1 Department of Microbiology and Genetics, Institute for Agribiotechnology Research (CIALE), University of Salamanca, 37185 Salamanca, Spain; me.morandiez@usal.es (M.E.M.-D.); belenru@usal.es (M.B.R.); rhp@usal.es (R.H.); emv@usal.es (E.M.); 2 Centre for Research and Technological Development of Water (CIDTA), University of Salamanca, 37080 Salamanca, Spain; jcgarcia@usal.es (J.C.G.-P.); mgr@usal.es (M.G.-R.); 3 Infectious and Tropical Diseases Research Group (e-INTRO), Biomedical Research Institute of Salamanca-Research Centre for Tropical Diseases at the University of Salamanca (IBSAL-CIETUS), Faculty of Pharmacy, University of Salamanca, 37007 Salamanca, Spain; juanbernalt95@usal.es (J.G.-B.D.); pfsoto@usal.es (P.F.-S.); ama@usal.es (A.M.); 4 FCC Aqualia, 28050 Madrid, Spain; esteban.serrano.leon@fcc.es (E.S.L.); victor.monsalvo@fcc.es (V.M.); mcasaom@fcc.es (M.C.)

**Keywords:** viral RNA, RT-LAMP, RT-qPCR, virus detection, wastewater-based epidemiology, sewage surveillance

## Abstract

Wastewater treatment plants (WWTPs) are the final stage of the anthropogenic water cycle where a wide range of chemical and biological markers of human activity can be found. In COVID-19 disease contexts, wastewater surveillance has been used to infer community trends based on viral abundance and SARS-CoV-2 RNA variant composition, which has served to anticipate and establish appropriate protocols to prevent potential viral outbreaks. Numerous studies worldwide have provided reliable and robust tools to detect and quantify SARS-CoV-2 RNA in wastewater, although due to the high dilution and degradation rate of the viral RNA in such samples, the detection limit of the pathogen has been a bottleneck for the proposed protocols so far. The current work provides a comprehensive and systematic study of the different parameters that may affect the detection of SARS-CoV-2 RNA in wastewater and hinder its quantification. The results obtained using synthetic viral RNA as a template allow us to consider that 10 genome copies per µL is the minimum RNA concentration that provides reliable and consistent values for the quantification of SARS-CoV-2 RNA. RT-qPCR analysis of wastewater samples collected at the WWTP in Salamanca (western Spain) and at six pumping stations in the city showed that below this threshold, positive results must be confirmed by sequencing to identify the specific viral sequence. This allowed us to find correlations between the SARS-CoV-2 RNA levels found in wastewater and the COVID-19 clinical data reported by health authorities. The close match between environmental and clinical data from the Salamanca case study has been confirmed by similar experimental approaches in four other cities in the same region. The present methodological approach reinforces the usefulness of wastewater-based epidemiology (WBE) studies in the face of future pandemic outbreaks.

## 1. Introduction

New emerging diseases continue to pose a major threat to human health. Coronavirus disease 2019 (COVID-19) broke out with great strength in our society, mainly due to the quick and easy transmission of SARS-CoV-2 through inhalation of aerosols/droplets and direct person-to-person contact [1,2]. Independent of the airborne and contact transmission of this virus and its localization in the respiratory tract and skin, SARS-CoV-2 RNA has been reported in urine [3] and faeces [4,5,6,7,8,9] of infected persons. In asymptomatic infected individuals with negative results for the presence of SARS-CoV-2 in nasopharyngeal swabs, the virus was readily detected over a 42-day period in their faecal samples [10]. The faeces, urine, and vomit of people infected with SARS-CoV-2 are discharged into sewage systems and subsequently pass into wastewater, allowing for at least the RNA from the viral particles to eventually reach wastewater treatment plants (WWTPs) [11]. This scenario prompted the idea that municipal wastewater from communities affected by the virus could contain traces of SARS-CoV-2 RNA, and different protocols and research approaches have been used to detect its genomic RNA in this context [12,13], making wastewater screening a major tool for population-wide surveillance of infectious diseases [14,15]. However, wastewater-based epidemiology (WBE), drawn from the initial idea that viruses could be shed into sewage in sufficiently high quantities to be detected, has generated some controversy, mainly due to the expected lability of both the virus itself and its RNA in such hostile environments [16,17]. Indeed, studies have shown that SARS-CoV-2 virions are degraded by the reactions that occur in WWTPs [18,19,20]. In any case, detection of SARS-CoV-2 genomic RNA traces in untreated wastewater was promptly reported in several countries, such as Australia [13], France [21], Japan [22], India [23], Italy [24,25], The Netherlands [19], Spain [18], Sweden [25], and the USA [26], in the early stages of the pandemic. It is accepted that sewage is not a putative route for COVID-19 transmission, but the fact that SARS-CoV-2 RNA was detected in wastewater led to some health concerns [27,28,29,30]. Comparison of viral genomic RNA copies with epidemiological data allows for the tracking of infection rates as an early warning WBE tool for prevention of COVID-19 disease outbreaks, informing decision making in public health programs [31].

SARS-CoV-2 RNA surveillance in wastewater is a complex approach engaging several stages until purified RNA is obtained for quantification [32,33]. The step of genome copy (gc) quantification normally relies on a reverse transcription–quantitative polymerase chain reaction (RT-qPCR) assay, considered the “gold-standard” method for RNA detection and quantification [34]. While it is likely that in most laboratories involved in SARS-CoV-2 RNA monitoring during the pandemic, inter-laboratory comparisons were performed to ensure accuracy of results, the huge amount of generated RT-qPCR data has been subject to the reliability and reproducibility of this methodology [35]. In this regard, the performance of a given primer set plays a key role in concentration determination by RT-qPCR assays, as it must be adjusted using the synthesis efficiency of a known template. 

In the present study, the limitations of the RT-qPCR technique for massive population testing of SARS-CoV-2 are examined. To this end, the efficiency of four described primer–probe sets targeting distinct regions of the viral genome that performed well on samples derived from infected patients was investigated for SARS-CoV-2 synthetic RNA quantification. Our experimental approach started from the idea that reagents work well when the template is at an optimal concentration but that they may not work as well when the template is diluted so that it becomes a limiting factor, which is a very plausible scenario in wastewater samples. Thus, after validating the correct functioning of two of the primer–probe sets at a concentration of only 10 gc µL^−1^, SARS-CoV-2 RNA was detected and quantified in wastewater samples from the Salamanca WWTP. Multiple samples collected over a period of eight months (September 2020 to April 2021) from seven different community facilities were analysed, resulting in a total sample set of 81 time points for the city of Salamanca. For WBE purposes, a pairwise comparison was performed using the obtained values and the clinical cases reported by the local health authority (https://analisis.datosabiertos.jcyl.es/pages/coronavirus/, accessed on 3 October 2023), defined as the cumulative number of new COVID-19 cases reported over 7 or 14 days. For comparison, SARS-CoV-2 synthetic RNA was also quantified, and SARS-CoV-2 RNA was detected and quantified in these wastewater samples by reverse transcription loop-mediated isothermal amplification (RT-LAMP). The results presented here demonstrate the obvious importance of the choice of primer–probe set, as well as the choice of concentration of template RNA minimally required to obtain reliable data in wastewater monitoring, which can be extended to other viruses similar to SARS-CoV-2. Despite the high degradation rate occurring in WWTPs, the trend of clinical cases in the city of Salamanca (Spain) and in four other nearby cities where our protocol was applied, shows the usefulness of wastewater monitoring of SARS-CoV-2 RNA to establish epidemiological alerts.

## 2. Results

### 2.1. Wastewater Sample Collection and Representativeness Evaluation

To achieve maximum representativeness of the monitoring sites, influent water samples were collected at the Salamanca WWTP and at six suburban pumping stations located within the metropolitan area of Salamanca encompassing ca. 170,000 inhabitants (Figure S1). The sampling point was determined at the inlet of the WWTP, and a preliminary 24 h study of an ordinary working day was carried out to determine the stretch of time during which the daily influent sample water should be taken. The determination of total coliform bacteria, as a recommended indicator for faecal contamination in wastewater, and TOC concentration served as indicator parameters (Figures S2 and S3) to determine the usual time slot of maximum evacuation of the population and, therefore, to explore the presence and predominance of SARS-CoV-2 viral traces in wastewater samples. As depicted in Figure S2, results of the hourly microbiological analysis of influent wastewater at the WWTP showed the highest value of coliforms at 11:00 h, whereas the turbidity reached the maximum at 17:00 h. A study of organic compounds determined that in the time interval of 10:00–18:00 h, there was a high TOC concentration, as shown in Figure S4, reinforcing the idea that around 11:00 h would be the best sampling window, as the viral RNAs are expected to be at their peak. Similarly, a study of Pearson correlations (significance level of 95% if *p* < 0.05) between variables was carried out (Figure S3), resulting in an inverse correlation (r = −0.2171 and *p* = 0.0473) between total coliforms and suspended solids to be precipitated (Figure S3A), while a positive correlation (r = 0.3067 and *p* = 0.0001) between the concentration of organic matter and suspended solids to be precipitated was also observed (Figure S3B). Moreover, there was no correlation (*p* > 0.05) between variables for faecal coliforms and suspended solids to be precipitated (Figure S3C). According to these results, 10–18 h was the time slot chosen for wastewater sample collection throughout the study.

### 2.2. Detection Limit Determined by RT-qPCR for SARS-CoV-2 RNA

**Figure 1:**
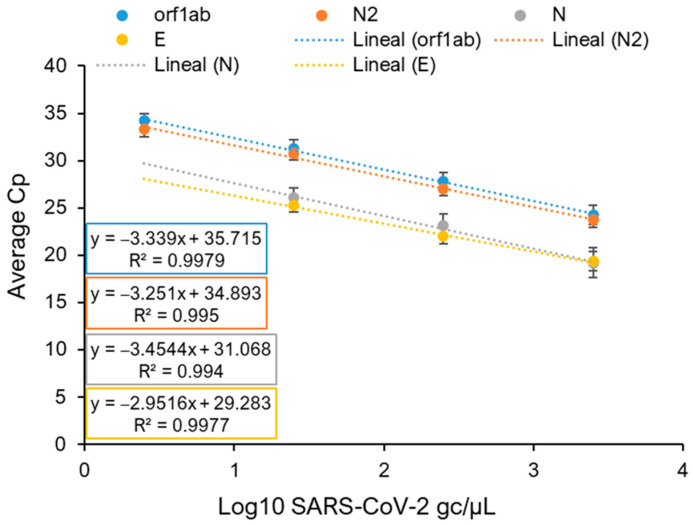
Validation of primer–probe sets using SARS-CoV-2 synthetic RNA. RT-qPCR was performed on viral genomic RNA (1 × 10^4^ to 1 × 10^1^ genome copies µL^−1^) to construct standard curves for the orf1ab, N2, N, and E regions to tabulate PCR amplification efficiency. Error bars show the standard deviation.

The use of synthetic RNA was chosen as the best option for RT-qPCR normalization. The SARS-CoV-2 synthetic RNA covers the entire ssRNA viral genome in six non-overlapping 5 kb fragments. The RNA is provided at a known concentration, which, after serial dilution, yields known concentrations ranging from 100,000 to 0.001 RNA gc µL^−1^ to be tested by RT-qPCR. In that way, standard curves are a suitable tool to interpolate SARS-CoV-2 RNA concentration from a given wastewater sample. Different oligo pairs targeting distinct regions of the viral RNA genome available in the literature (Table S1) were tested. It must be considered that when amplifying the reverse-transcribed cDNA from viral RNA using the previously described primer–probe sets, a single amplicon was not always obtained, as determined by melt curve analysis. For instance, using the primer pair of E_Sarbeco_F1 and E_Sarbeco_R2 (Table S1) targeted to the E gene, the melting curve showed two peaks—a result that is usually interpreted as primer dimers (Figure S5A). In fact, follow-up analysis by gel electrophoresis (Figure S5B,C) revealed that both curves generated two discrete amplicons, confirming that a single PCR product was not obtained. For RT-qPCR reactions aimed at generating the standard curve, the concentrations in problem samples ranged from 25,000 to 0.0025 gc of synthetic RNA per µL. It was observed that the obtained values were not reliable for SARS-CoV-2 synthetic RNA concentrations below 1.0 gc µL^−1^ and that lower values imply pipetting errors, which have been shown to have a major impact on data consistency. High variability among technical replicates was observed for concentrations below 10 gc µL^−1^ in the standard curve (that is, below 2.5 gc per µL in a 20 µL reaction) (Figure S6). Furthermore, apart from problems generated by the primer–probe set targeting the E-coding region, the primer–probe set directed to N in 5’-coding region also yielded ambiguous results among technical replicates at the standard curve point of 10 gc µL^−1^ (Table S2). Thus, this concentration was established as the lowest point of the standard curve, with 100, 1000, and 10,000 gc µL^−1^ being the concentrations chosen to generate the standard curve. As the Cp obtained for each point varies from one plate to another, it was necessary to include the four points, with three technical replicates for each of them, in each plate that was made. In that way, viral gc could be calculated based on the Cp obtained in the problem sample (Figure 1). Moreover, our results indicate that, out of the four tested primer–probe sets, only those targeting orf1ab and N in 3′-coding regions showed reliable performance at a concentration of 10 gc µL^−1^ on the standard curve. Thus, these two effective primer–probe sets were elected as trustworthy tools for subsequent analysis of wastewater samples.

To assess the extent to which the way cDNA was synthesised had an impact on the detection and quantification of SARS-CoV-2 RNA, a two-step approach was further evaluated. To do so, RT was performed in a pre-qPCR reaction using random hexamers or specific primers for cDNA synthesis, as well as undiluted SARS-CoV-2 synthetic RNA. Similar to the results obtained when amplifying SARS-CoV-2 synthetic RNA in a one-step assay, a high variability among technical replicates was detected for standard curve point concentrations below 10 gc µL^−1^ (that is, below 2.5 gc µL^−1^ in a 20 µL reaction). Still, the Cp values obtained by the two approaches were quite similar for each concentration point. Although it is not feasible to obtain a linear correlation, the fact that the Cp for each concentration point was in the same range showed that performing RT and qPCR reactions in a single tube using the one-step approach was not only appropriate but the best option for further analysis of wastewater.

In addition, the RT-qPCR amplicons obtained at each point of the standard curve were sequenced to confirm and estimate the percentage of real amplification of the viral genome. In early sampling, PCR amplicons were used directly for sequencing after purification but with little success, since in most cases, no sequence was retrieved. To overcome this problem, which could be due to the generation of a mixture of amplicons during the PCR reaction, amplicons were cloned before sequencing. The sequence of the SARS-CoV-2 control was straightforwardly detected with up to 100% success when 10,000 gc µL^−1^ standard curve point PCR products were analysed (Table 1). However, the percentage decreased as the analysed concentration of the standard points decreased, reaching the lowest value of 40% when the one-step RT-qPCR assay was performed using the primer–probe set targeting the orf1ab-coding region for the 10 gc µL^−1^ standard curve point (Table 1). RT-qPCR performance was much worse when two-step assay amplification products were sequenced; in this case, targeting N in the 3′-coding region for the 10 gc µL^−1^ standard curve point achieved 0% success (Table 1). Similarly, to confirm that the PCR products generated in positive RT-qPCR wastewater samples corresponded to the expected regions of the viral genome, amplicons were sequenced. As for the standard curve points, the correct sequence of SARS-CoV-2 was directly detected, confirming that the analysed samples contained genomic viral traces. Nonetheless, by-products that share homology with another organism other than SARS-CoV-2 were sequenced. In the case of the sample collected on 27 October 2020, 50% of the amplicons sequenced when targeting the orf1ab-coding region shared homology with *Acinetobacter* spp.; on that day, the gc of SARS-CoV-2 RNA was calculated to be 1.16 × 10^4^ gc L^−1^ using the same primer–probe set. Likewise, 50% of the amplicons sequenced when the orfab1-coding region was targeted in the sample collected on 10 November 2020 showed no homology with sequences present in the databases, the calculated gc of SARS-CoV-2 RNA on that day being 2.29 × 10^4^ gc L^−1^ using the same primer–probe set. Thus, sequenced by-products that either shared homology with organisms other than SARS-CoV-2 or did not show homology with sequences present in the databases were considered PCR artifacts.

### 2.3. Detection Limit Determined by One-Step RT-LAMP for SARS-CoV-2 RNA

**Figure 2:**
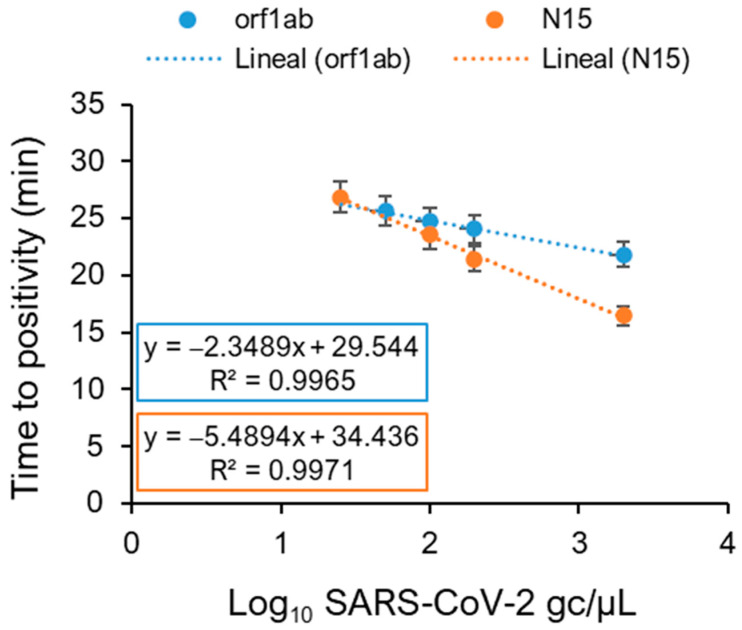
Validation of primer sets using SARS-CoV-2 synthetic RNA. Real-time RT-LAMP assays were performed on viral genomic RNA (1 × 10^4^ to 1 × 10^1^ genome copies µL^−1^) to construct curves for the orf1ab and N15 regions to tabulate PCR amplification efficiency. Time to positivity (Tp) for each primer set is indicated. Error bars show the standard deviation.

As for RT-qPCR reactions, different SARS-CoV-2 synthetic RNA concentrations (1000, 100, 50, 25, 12.5, and 10 RNA gc µL^−1^) were used to generate the RT-LAMP standard curves. For each concentration point, four viral regions were tested (Figure S7), and their time-to-positivity (Tp) robustness was determined by including three technical replicates. A high variability among regions was observed when analysing the performance of primer sets for SARS-CoV-2 RNA amplification. For instance, the primer set targeting the E-coding region was non reliable enough for detecting standard curve points less than 2000 gc µL^−1^ (that is, 266.66 gc µL^−1^ in a 15 µL reaction). Similarly, targeting N in the 5-coding achieved negative results for concentrations below 100 gc µL^−1^ (that is, 13.33 gc µL^−1^ in a 15 µL reaction). On the other hand, consistent results were obtained when orf1ab- and N15-coding regions were targeted, achieving positive results at a concentration of 50 gc µL^−1^ (that is 6.66 gc µL^−1^ in a 15 µL reaction). Eventually, the primer set targeting the N15-coding region achieved positive results in some reactions at a concentration of 25 gc µL^−1^ (that is, 3.33 gc µL^−1^ in a 15 µL reaction). Thus, orf1ab and N15 primer sets were selected as reliable tools for subsequent analysis of wastewater samples. SARS-CoV-2 RNA detection was not achieved by RT-LAMP for concentrations below 1.5 gc µL^−1^ per reaction (Figure 2 and Table S1). Moreover, the 50 gc µL^−1^ standard curve point concentration was established as the lowest standard curve point, with the other points for generating the standard curve being 100, 1000, and 10,000 gc µL^−1^. As occurred for RT-qPCR, the Tp obtained for each point varied from one plate to another, so it was necessary to include the four points with three technical replicates for each of them in every reaction plate. In that way, the viral gc could be calculated from the Tp obtained in the problem sample.

### 2.4. Effect of the Wastewater Environment on RNA Stability

Since samples from wastewater may contain environmental inhibitors that could affect RT-qPCR, a specific commercially available silica membrane mini spin column kit was chosen for viral RNA extraction from concentrated extracts. In this way, contaminants and enzyme inhibitors that could lead to false negatives could be removed. The yield of RNA recovered after RNA isolation could not be determined, as carrier RNA was used during the RNA extraction procedure. Specific primers and probes were chosen to target at least two regions of the viral genome. To evaluate how RNA could be altered in such adverse conditions, a non-targeted RNA sequence was added in few concentrated wastewater samples as an exogenous spiked-in control. Total RNA extracted from PSTVd-infected plants was added to the concentrated samples prior to performing the RNA extraction. Viroid RNA is a very structured kind of RNA that, when compared to mRNA, allows for discernment of the ratio of degradation based on the folding properties of the RNA. Recovery of plant viroid RNA was so low that in some samples, 96% was lost, giving an idea of how unfriendly this environment is to a small circular naked RNA molecule and how much more hostile it is to larger, less structured RNA.

### 2.5. Wastewater SARS-CoV-2 Detection and Effectiveness for Epidemiology Monitoring

**Figure 3:**
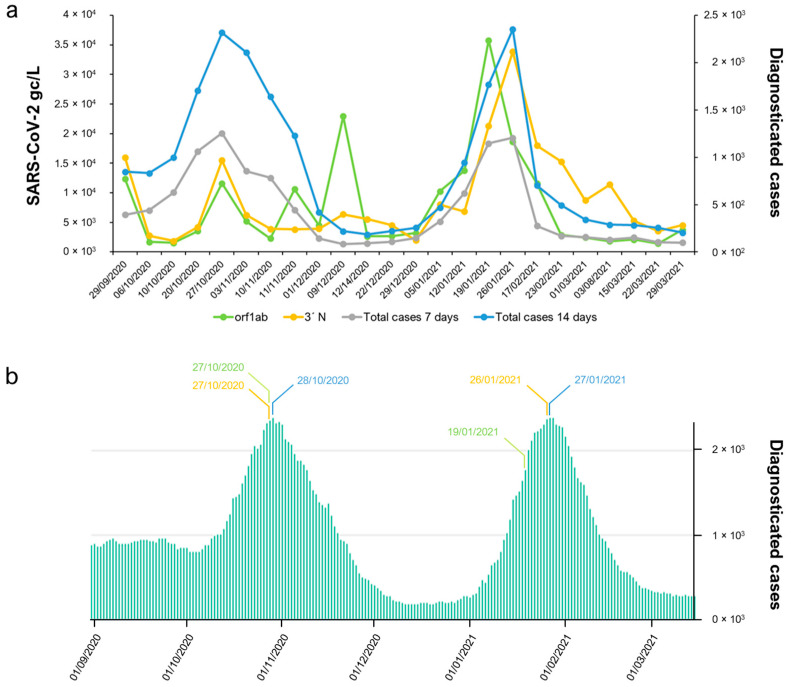
Evolution of the presence of the SARS-CoV-2 genome in Salamanca’s wastewater treatment plant (WWTP). (**a**) Calculated gc for the RNA-dependent RNA polymerase using a primer–probe set targeting the orf1a viral coding region and the nucleoprotein RNA employing a primer–probe set targeting N in 3′-coding regions, along with reported COVID-19 cases. Values represented on the left axis refer to the estimated gc mL^−1^ of collected samples, while values represented on the right axis denote the cumulative incidence of cases diagnosed in 7 or 14 days provided by the local health authority (https://analisis.datosabiertos.jcyl.es/pages/coronavirus/, accessed on 3 October 2023). (**b**) Representation of the everyday cumulative incidence of cases diagnosed in 14 days provided by the local health authority during the analysed period. Days with the maximum number of reported cases, days with the maximum gc mL^−1^ calculated for the orf1ab-region, and days with the maximum gc mL^−1^ calculated for the N-coding region are marked in blue, green, and orange, respectively.

**Figure 4:**
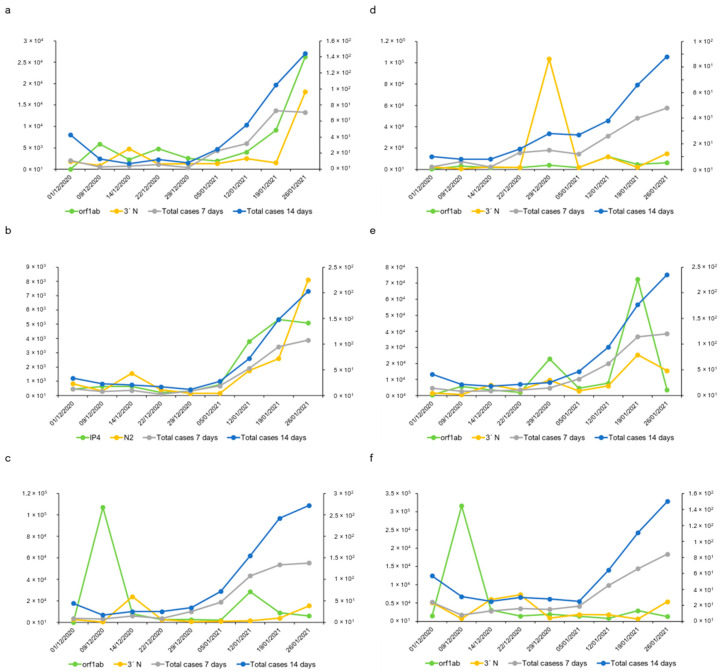
Evolution of SARS-CoV-2 genome copy levels in samples collected from six urban sewers in Salamanca. (**a**) San José; (**b**) University district; (**c**) San Bernardo; (**d**) Capuchinos; (**e**) hospital; (**f**) nursing home. Values represented on the left axis refer to the estimated gc mL^−1^ of collected samples, while values on the right axis denote the cumulative incidence of cases diagnosed in 7 or 14 days provided by the local health authority (https://analisis.datosabiertos.jcyl.es/pages/coronavirus/, accessed on 3 October 2023).

The comparative sensitivity of the RT-qPCR and RT-LAMP techniques (Figure 1 and Figure 2) showed differences when employing a SARS-CoV-2 synthetic RNA standard as a template. Furthermore, each technique revealed that, depending on the primer set used to amplify the SARS-CoV-2 synthetic RNA, differences could be observed (Figure 1 and Figure 2). Positive results were obtained by RT-LAMP with concentrations of 6.66 gc µL^−1^ per reaction when orf1ab- and N15-coding regions were targeted (Figure 2). As no detection was achieved for RT-qPCR concentrations below 1.5 gc µL^−1^ per reaction, its detection limit was set at 2.5 gc µL^−1^ per reaction for orf1ab- and 3′N-coding regions (Figure 1), corresponding to a difference of just over 2.5 fold in the limit of detection in favour of RT-qPCR. Results consistent with the clinical data on the incidence of COVID-19 in the city of Salamanca were obtained (Figure 3 and Figure 4) when the presence of SARS-CoV-2 RNA in wastewater was quantified by either technique.

**Figure 5:**
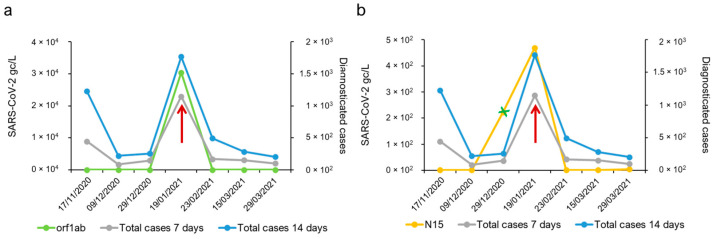
RT-LAMP analysis of RNA extracted from sewage water. (**a**) Detection of the RNA-dependent RNA polymerase RNA using orf1ab primers. (**b**) Detection of nucleoprotein RNA employing N15 primers. The green line denotes the estimated number of viral RNA genome copies per L of wastewater sample, while the black and pink lines denote the cumulative incidences of cases diagnosed in 7 and 14 days, respectively. A positive sample collected on 19 January 2021 is identified by a red arrow. The green X denotes samples for which not all replicates were positive. Any sample with a time to positivity (Tp) over 40 min was considered negative.

The highest cumulative number of new reported COVID-19 cases over 7 or 14 days within the period analysed in the current study was reported on 27 January 2021 (Figure 3), corresponding to 1.21 × 10^3^ and 2.35 × 10^3^ cases, respectively. The gc calculated by the RT-LAMP assay reached its maximum on 26 January 2021, independent of the used primer–probe set. The estimated gc according to the RT-LAMP analysis as 1.64 × 10^4^ gc µL^−1^ when the orf1ab-coding region was targeted, while the gc estimated using the same RNA sample as the template dropped to 1.51 × 10^2^ gc µL^−1^ when the N15-coding region was targeted (Figure 5). Still, both primer sets achieved the maximum gc just one day ahead of the cases reported by the local health authority. Similarly, results obtained by the RT-qPCR resulted in maximum gc on 26 January 2021, with a value of 1.36 × 10^4^ gc µL^−1^ when N was targeted in the 3′-coding region (Figure 3). However, when the orf1ab-coding region was targeted by RT-qPCR assay, the maximum cg of 3.58 × 10^4^ gc µL^−1^ was obtained on 19 January 2021, one week ahead of the maximum gc calculated by the other RT-qPCR primer set used, as well as the one calculated by RT-LAMP, and 8 days ahead of the highest cumulative number of new reported COVID-19 cases. The study was extended to four other locations in the Castile and Leon regions with different population sizes, economic activities, and incidences of COVID-19. Hence, wastewater samples collected at the Ávila, Guijuelo, Aranda de Duero, and Medina del Campo WWTPs were analysed. Still, regardless of the heterogenicity related to the two parameters mentioned above, a good correlation was obtained between clinical data and SARS-CoV-2 viral RNA quantification (Figure S8), demonstrating the reliability of the parameters chosen for the analysis. The detection of SARS-CoV-2 in naturally contaminated wastewater revealed that 100% positive detection results were obtained in all tested wastewater samples, and viral RNA was readily detected.

## 3. Discussion

The approach based on monitoring and examining wastewater to gain insights into the health communities has been used for decades [36]. Nevertheless, WBE has gained significant attention, particularly during the COVID-19 pandemic, as a means to observe the prevalence and spread of infectious diseases. Indeed, the use of WBE as a community surveillance tool has enabled non-invasive and mass localized screening for SARS-CoV-2 outbreaks and has been widely accepted and embraced by many countries [37]. In most individuals, SARS-CoV-2 detection in clinical tests is performed when patients become symptomatic, making it difficult to obtain a comprehensive or high-level view of trends in a community or infection rates based on clinical data alone. However, it is possible that a patient may transmit the virus before becoming symptomatic or that a patient may be infected but symptomless for the duration of the infection. Therefore, wastewater surveillance, especially at regular sample sites ranging from WWTPs to more granular locations including hospitals, schools, individual neighbourhoods, and high-risk buildings such as long-term care facilities, has become a powerful tool for addressing the epidemiological situation of a population and for decision making [38], especially given that the RNA associated with the presence of SARS-CoV-2 has been able to be detected 6 days before the first clinical cases are reported in a population [19,39].

The presence of faecal coliform bacteria in water provides information regarding stool contamination in warm-blooded animals [40], but it can also serve to estimate the load of viral debris excreted with faeces [41]. This correlation allowed us to calculate that the best time for sample collection was at 11:00 am, at least in the WWTPs included in our study during the COVID-19 outbreak. However, human waste mixes with domestic, industrial, and drainage water, and viral traces can be found at low concentrations in raw sewage, making it necessary to concentrate the samples prior to analysis [42]. There is no consensus on the best concentration method for reliable detection of SARS-CoV-2 in wastewater, considering differences in laboratory facilities. For instance, when comparing ultracentrifugation and skimmed-milk flocculation concentration methods, the detection limits ranged from 1.86 × 10^3^ to 1.26 × 10^7^ gc µL^−1^ between the former and the latter methodology [43]. In our case, the samples were first concentrated by a simple and previously validated chemical coagulation–flocculation concentration protocol [18,44].

RT-qPCR is the “gold-standard” method for clinical testing for SARS-CoV-2 [34], and it has proven useful for the study of the presence of SARS-CoV-2 RNA fragments in wastewater [45,46,47]. Multiple nucleic acid amplification tests targeting different SARS-CoV-2-coding regions have been developed to detect RNA of this virus in COVID-19 patients, and different sensitivities have been reported [38]. Obviously, the viral load in clinical samples is much higher than that to be expected in wastewater samples; thus performance validation of primer–probe sets is a required step to avoid the generation of erroneous trends when the viral concentration is below a certain level. Moreover, many studies have made use of surrogate viruses that structurally and morphologically resemble SARS-CoV-2 [38]. As no strong epidemiological evidence has been found to support human faecal–oral transmission of SARS-CoV-2 [27,30] and in view of the environmental conditions offered by sewage to infectious viral particles, it is very clear that the presence of the whole virus is not detected in wastewater samples [28]. Attempts to obtain cell lines infected by virions presumably present in wastewater failed [48], and this is probative of why the handling of this type of sample can be considered safe, with no infectivity test usually performed. Similarly, many laboratories have quantified the gc of the virus by plotting the amplification cycles to an external standard curve built with plasmid DNA, providing an extra parameter that can compromise the calculated gc, since the RT step performed for SARS-CoV-2 quantification is omitted in the generation of the standard curve [38].

In this study, the RT-qPCR detection limit was established by employing SARS-CoV-2 synthetic RNA to build the standard curve on which amplification cycles could be plotted for SARS-CoV-2 gc quantification. Furthermore, exogenous RNA was spiked in as a quality control to identify RT-qPCR inhibitors and analyse recovery rates. Finally, after comparing one-step and two-step performance, samples collected from WWTPs and sewer sheds were analysed by a one-step approach, allowing for efficient synthesis of cDNA and qPCR in a single tube. Other WBE methodologies have been used for the detection of SARS-CoV-2 in wastewater samples [49], such as next-generation sequencing (NGS) techniques, which have proven to be extremely useful for the identification of specific SARS-CoV-2 variants [50,51,52,53], but not all laboratories have sufficient assets to employ this methodology on a regular basis. This type of approach is also time-consuming, limiting the number of samples that can be processed. The same is true for ultrasensitive assays based on RT-qPCR such as droplet digital PCR (ddPCR) [54]. Despite providing a direct quantification without the need for external calibration, ddPCR, like NGS, requires not only specific and extensive instrumentation but high-cost reagents, which makes it expensive, unaffordable for regular use, and (at least in our case) not available in the WWTP laboratories responsible for potential WBE analysis. RT-LAMP is a less equipment-dependent technique that has proven to be reliable for the detection of SARS-CoV-2 in wastewater [55,56]. And, like RT-qPCR, it is simple and fast (1 h, approximately). Due to the exponential amplification feature of RT-LAMP, four different primers can detect six different target sequences at the same time, and at the end point of detection by this technique, the amplified products are subsequently analysed by gel electrophoresis [57], although they cannot be sequenced. Sequencing methods are employed for confirmation of PCR products. Something that can be done on a regular basis in order to avoid working with artifacts and, as mentioned above, owing to the peculiar complexity of wastewater, may easily generate quite false positives. Indeed, given the pressure of working during the pandemic, not many research groups [22,23,24,48,58,59] decided to apply this methodological step aimed at sorting out PCR-derived by-products that could interfere not only with the correct viral detection but with also with its precise quantification. In the current study, the sensitivity of RT-LAMP and RT-qPCR on the same samples was compared (collected from 17 November 2020, to 6 April 2021) to evaluate the relationality of the quantified copies of genomic RNA traces per mL of wastewater sample. Despite the higher speed of RT-LAMP, according to previous reports [60], a lower detection limit was found for RT-qPCR. 

The amplification efficiency of RT-qPCR was close to 100% in most runs (normally, desired amplification efficiencies ranged from 90 to 110%), and the theoretical maximum of 100% indicates that the polymerase enzyme was working at maximum capacity, which indicates perfect efficiency. Nevertheless, it was reported that small changes in PCR efficiency from 100 to 97% over 30 cycles caused a 57% difference in the estimated input DNA, while a change from 100 to 90% resulted in a 36.5% difference [61]. This is why the origin of the sample and the nature of the viral RNA molecule within may play a key role in the standardization of protocols and that this may imply a different choice of target region in the viral genome. SARS-CoV-2 RNA copies were found in varying amounts in wastewater samples from Australia (3.1 × 10^3^ and 7.5 × 10^3^ gc L^−1^) [26], France (5 × 10^4^ and 3 × 10^6^ gc L^−1^) [21], and Salamanca (3.84 × 10^3^ and 1.06 × 10^4^ gc L^−1^, sample collected on 11 November 2020, in this study). Since it is mandatory to design suitable primer pairs because viruses can go undetected if they are improperly designed or if the virus has mutated in the target genome region, traces of the virus may be sufficient for its proper detection in the peculiar environment of wastewater and, thus, potentially useful for viral load quantification [62]. Therefore, it should be noted that not all primer–probe sets can be used to adequately quantify the viral RNA copy number in a given sample. Even so, they can be used to determine the presence/absence of viral genomic material in wastewater samples. Primer–probe sets that have been proven to perform correctly on clinical samples can generate amplicons for which up to 50% were artifacts when template concentrations were low, which is the norm prior to an outbreak alert, so detection and quantification can be biased due to increased artifacts, irrespective of the Cp values. As for confirmation of virus variants, the sequence of the amplicons is needed to identify false positives. In our case, when using too low RNA template concentrations, something that, in real samples, would not be possible to know, a Cp value of 34 was obtained for the lowest point of the standard curve (Table S2). Our study shows that many amplifications were basically artifacts—up to 60% for the lowest point of the standard curve (Table 1). Thus, artifact amplification could interfere not only in the correct detection of the virus but also in its accurate quantification, since false-positive results were generated. This is a very sensitive point because in the first analyses carried out on wastewater samples during and following the COVID-19 pandemic outbreak, the motivation and the desire that the developed SARS-CoV-2 detection methodologies be effective and have a real utility, even passing the evaluation filters of the multiple projects that were funded specifically for this purpose, led to different research groups, including ours, being questioned in the face of the wave of positivity that was being offered by many laboratories [63]. In the hostile environment of WWTPs for viruses, sequencing of PCR products to confirm that they belong to amplified fragments of the target virus and that they are not artifacts is a mandatory step [64], a procedure confirmed by the correlation of our data with the epidemiological curves in the population published by the health authorities (Figure 3, Figure 4 and Figure 5). A sequencing approach similar to that of other laboratories [13,22,23,24,48,58,59] was followed as a final step in the identification of RNA from SARS-CoV-2 in wastewater. Knowledge of the amplicon sequence obtained by using control RNA as a template not only allowed us to determine the lowest concentration rate that provided robust results but facilitated information about the error that could be introduced when quantifying the virus titre.

There is an ongoing debate about how wastewater monitoring data and clinical infection rates should be used in combination and the implications they may have for public health decision making [47], for instance, how disease trends and the effectiveness of disease control measures taken in a community could be inferred. Some studies have reported a positive correlation between hospitalizations of infected individuals presenting symptoms due to COVID-19 with the quantification of SARS-CoV-2 RNA concentration in wastewater collected at WWTPs [36]. However, the total viral gc also includes viral RNA from asymptomatic individuals, with an inherent bias to the viral load of each individual [19]. In our study, the sampling of five WWTPs and six suburban pumping stations was coordinated for a frequency of once a week. According to recent work [65], a frequency of less than three times per week may lead to unreliable estimates of the effective reproduction number (*R_t_*) based on wastewater monitoring data alone. Still, it was pretty surprising that the overall result derived from wastewater testing and clinical data matched so closely in the city of Salamanca and in four other cities in the same region but with very different economic activities, namely Ávila (tourist), Guijuelo (livestock production and transformation), Aranda de Duero (industry), and Medina del Campo (agriculture) (Figure S8). However, in view of recent publications on the subject, if the work were to be carried out again, it seems more advisable to ensure the results with a higher weekly sampling frequency to avoid the risk of obtaining unreliable data for *R_t_* calculation. It should also not be ruled out that the correlation between viral RNA quantification and clinical data may be not only due to the non-consideration of false positives eliminated after amplicon sequencing but also to the handled volumes, a precise sample concentration step, and the RT-qPCR conditions, including the selection of suitable primers.

## 4. Materials and Methods

### 4.1. Sampling Locations and Collection of Wastewater Samples

A total of 27 untreated wastewater samples (1 L each) were collected almost weekly from 28 September 2020 to 19 April 2021 at the WWTP of the city of Salamanca (western Spain) and at six suburban pumping stations located within its metropolitan area. The six suburban points were located in the neighbourhoods of San José, the University district, San Bernardo, and Capuchinos, as well as the central hospital and a nursing home (Figure S1). Wastewater samples were also collected in four other cities in the same region with different types of economy, namely Ávila (tourism and services), Guijuelo (livestock production and transformation), Aranda de Duero (food and pharmaceutical industries, automotive components, and infrastructure), and Medina del Campo (agriculture). The samples from the WWTP were collected from the influent, and those from the suburban pumping stations were taken from the sewers, which were considered, in all cases, raw wastewater. The time slot (10:00 h–18:00 h) was chosen from 24 h of data according to the information obtained from previous bacteriological analysis aimed at determining the coliform bacteria content. To generate the samples, 1 L of composite sample was collected every hour from 10:00 h to 18:00 h and added to a 10 L container, generating an 8 L sample that was kept at room temperature, avoiding solar exposure in the sampling location. After shaking, a 1 L aliquot was taken. All samples were collected in sterile plastic bottles and immediately transported to the laboratory. During transportation, the samples were kept refrigerated and at 4 °C in the laboratory until 200 mL from each sample was processed, usually within 24 h.

### 4.2. Bacteriological Analysis

All analyses were carried out aseptically, and the standard methods described by the American Public Health Association [66] were followed. Bacteriological analysis of the wastewater samples from each collection point was performed to determine the presence of coliform bacteria, and colony-forming units (CFUs) were used to estimate the number of viable bacteria with the pour-plate method [66] on chromogenic agar (Scharlab, Sentmenat, Spain). Wastewater turbidity was also determined in all collected samples by employing a portable turbidity meter (Hach Company, Loveland, CO, USA) and expressed in nephelometric turbidity units (NTUs) (Figure S2). For the calculation of total organic carbon (TOC), total coliform (TC), and injured coliform (IC), a filtration method employing a fiberglass filter was employed in accordance with Spanish standard UNE-EN 872:2006 for water quality regarding the determination of suspended solids in raw, waste, and treated water (Figure S3). Organic matter was determined by measuring the 680 °C combustion catalytic oxidation with a non-dispersive infrared detector (NDIR) on a Shimadzu TOC 5000-A (Shimadzu Corporation, Kyoto, Japan) (Figure S4).

### 4.3. Preparation of Wastewater Samples for RNA Extraction

The Spanish Government, through the VATar COVID-19 project, provided a standard protocol for the detection of SARS-CoV-2 in wastewater (https://www.miteco.gob.es/content/dam/miteco/es/agua/temas/concesiones-y-autorizaciones/vertidos-de-aguas-residuales/alerta-temprana-covid19/protocolo-deteccion-sars-cov-2-en-aguas-residuales_tcm30-528265.pdf, accessed on 3 October 2023) that considers both personnel and laboratory equipment safety practices in accordance with the World Health Organization [67] laboratory biosafety manual. The protocol establishes procedures for sampling, shipping, and receiving, as well as concentration and extraction of wastewater samples. Therefore, 200 mL was treated from each wastewater sample, and RNA was extracted following the mentioned standardized protocol. In a reduced number of wastewater samples, a tittered preparation of RNA extracted from tomato plants infected with potato spindle tuber viroid (PSTVd) was spiked into concentrated wastewater samples to serve as an exogenous control to monitor recovery efficiency after RNA extraction. For that purpose, 1 μL of plant RNA at a concentration of 1.14 μg μL^−1^ was added to 200 mL of wastewater samples.

### 4.4. RNA Detection Methodologies and Standard Curve Optimization

RNA extraction and amplification analyses were performed in different rooms to avoid any contamination. RT-qPCR assays were performed using two approaches, namely a separate RT reaction followed by qPCR (two steps) and a combined RT and qPCR reaction in the same tube (one step). For the one-step assay, RT-qPCR analyses were performed in 20 µL reaction mixtures using TaqMan Fast Virus 1-Step Master Mix (Applied Biosystems, Foster City, CA, USA). RT-qPCR mixtures contained 5 µL of 4X TaqMan Fast Virus 1-Step Master Mix, 1.8 µL of 10 µM forward primer, 1.8 µL of 10 µM reverse primer, 0.4 µL of 10 µM TaqMan probe, 6 µL of RNase-free MilliQ water, and 5 µL of template RNA. This amount of RNA corresponds to 20 mL of the initial volume of wastewater sample. For the two-step assay, a PrimeScript 1st strand cDNA Synthesis kit (Takara Inc., Tokyo, Japan) was used either with random hexamers or specific primers (Table S1) for cDNA synthesis. In such cases, RT reactions were performed in 10 µL, and each reaction mixture contained 2 µL 5X PrimeScript Buffer, 0.5 µL PrimeScript RT Enzyme Mix I, 0.5 µL 100 µM random hexamers or 0.5 µL 50 µM specific primer, 2 µL RNase-free MilliQ water, and 5 µL template RNA, following the manufacturer’s indications. Then, 40 µL MilliQ water was added to the RT reaction prior to qPCR amplifications, which were performed using 2 µL of synthesised cDNA as described for the one-step method. Alternatively, reactions were performed in a total volume of 10 µL, using SYBR FAST KAPA qPCR (Biosystems, Buenos Aires, Argentina). qPCR mixtures contained 5 µL 2X Fast SYBR Green Master Mix, 0.2 µL 10 µM forward primer, 0.2 µL 10 µM reverse primer, and 2.6 µL MilliQ water. All qPCR reactions were performed on a StepOne Plus device (Applied Biosystems, Waltham, MA, USA), and three biological replicates for each wastewater sample were analysed in triplicate. For SARS-CoV-2 genomic RNA detection and quantification assays, hydrolysis probes were used (Table S1). The thermal RT-qPCR cycling parameters were 50 °C for 5 min and 95 °C 20 s followed by 40 cycles of amplification at 95 °C for 3 s and 60 °C for 30 s, omitting the first step at 50 °C for 5 min when qPCR reactions were performed on cDNA templates.

Making use of SARS-CoV-2 synthetic RNAs (Twist Bioscience, San Francisco, CA, USA) that cover the entire ssRNA viral genome in 6 non-overlapping 5 kb fragments (accession numbers MT007544.1 and MN908947.3), a series of standard curves was generated for absolute quantification of viral gc and in order to determine the detection limit for each evaluated primer–probe set targeting orf1ab, E, and N in the 5′-coding regions, and N in the 3′-coding regions (Table S2). These standards were supplied at a known concentration of 1,000,000 (10^6^) gc µL^−1^ (17 pg µL^−1^). The synthetic RNA was serially diluted to obtain dilutions to be tested by RT-qPCR ranging from 100,000 (10^5^) to 0.001 (10^−3^) gc µL^−1^. Reactions generated undetermined crossing points (Cp), or number of cycles at which the amplification plot crosses the threshold (value above the background) when the RNA concentration was below 10 gc µL^−1^ (170 ag µL^−1^). Thus, for each RT-qPCR run, four concentrations of a 10-fold serial dilution of the quantified SARS-CoV-2 RNA control were used, ranging from 1 × 10^4^ to 1 × 10^1^ gc µL^−1^, and no template (RNase free water) controls were included. Then, 5 µL of Twist SARS-CoV-2 synthetic RNA control was used in a 20 µL reaction, and RNA concentrations for the four positive samples ranged from 2500 to 2.5 gc µL^−1^. Those four points were used to generate the standard curves in each run. RT-qPCR Cp data obtained for each run were plotted on the standard curve produced in the same run for SARS-CoV-2 RNA sample quantification as gc. Alternatively, SARS-CoV-2 RNA-derived cDNA was synthesised using 7 µL of the quantified synthetic RNA template (1 × 10^6^ gc µL^−1^) and a PrimeScript 1st strand cDNA Synthesis Kit (Takara Inc., Tokyo, Japan), employing either random hexamers or specific primers. Then, a 10-fold serial dilution of synthesised cDNA ranging from 1 × 10^4^ to 1 × 10^1^ gc per µL was used for qPCR analysis using TaqMan Fast Virus 1-Step Master Mix as described above.

To detect SARS-CoV-2 genomic RNA using one-step RT-LAMP, the orf1ab-, E-, N5-, and N15-coding regions (Figure S7) of the SARS-CoV-2 genome were targeted, and assays were performed as previously described [68] using 2 μL RNA template or RNase-free MilliQ water for no template samples. Similarly, as for RT-qPCR, three biological replicates for each wastewater sample were analysed by RT-LAMP in triplicate. The reaction protocol was 40 min at 63 °C followed by a 1 min inhibition step at 95 °C. As for RT-qPCR, a standard curve for absolute quantification of viral gc was generated with synthetic RNA standards of the SARS-CoV-2 genome. The synthetic RNA was serially diluted at 1 × 10^3^, 1 × 10^2^, 5 × 10^1^, 2.5 × 10^1^, and 1.25 × 10^1^ gc µL^−1^, and 5 µL was used in a 20 µL RT-LAMP reaction. As for RT-qPCR, those points generated standard curves on which the data obtained in each run could be plotted for the quantification of SARS-CoV-2 RNA samples as gc.

### 4.5. Sequence Determination of Generated Amplicons

Amplified products were electrophoresed on 1% agarose gels buffered with 0.5 X TBE (89 mM Tris, 89 mM boric acid, and 2 mM EDTA; pH8) and stained with ethidium bromide. The amplified PCR products were excised from the gels, and DNA was purified using a NucleoSpin Gel and PCR clean-up kit (Macherey-Nagel GmbH & Co., Düren, Germany) according to the manufacturer’s instructions. Once purified, amplicons were cloned into the pCR 2.1-TOPO vector using a TOPO TA Cloning kit (Invitrogen, ThermoFisher Scientific, Carlsbad, CA, USA) and sequenced employing the M13 forward and M13 reverse primers (Table S1) on a 3100 Genetic Analyzer (Applied Biosystems, Waltham, MA, USA) on the Nucleus platform of the University of Salamanca (Salamanca, Spain). Obtained sequences were subjected to homology determination using the BLAST database tool (http://blast.ncbi.nlm.nih.gov/Blast.cgi, accessed on 3 October 2023).

### 4.6. Statistical Analysis

Data obtained from the bacteriological and organic carbon analyses were subjected to statistical analysis using SIMFIT statistical package version 7.3.1 (Bardsley W.G 2017 Simfit Statistical package. v. 7.3.1 Academic 64-bit Manchester University, Manchester, UK). Geometric means of bacterial counts were computed, and analysis of Pearson correspondences between variables was carried out using the correlation module of the SIMFIT statistical package to determine differences in bacterial count from various sampling points at a 95% level of significance.

## 5. Conclusions

The COVID-19 pandemic has taught us that WBE is useful for public health surveillance for future disease outbreaks caused by viruses or other infectious microorganisms, as the possibility of detecting early warning signals through wastewater data can allow us to make appropriate public health decisions based on observed case trends in a community. The opportunity to establish differences in wastewater loads by area within a population provides epidemiological data on specific points (hospitals, nursing homes, schools, etc.) in terms of what is happening in the community. The present study also serves to corroborate that the robustness of WBE analysis depends, to a large extent, on the chosen approach and the methodology described to carry it out, while, in addition to reporting a case study of the different parameters that need to be assessed to generate an appropriate protocol for SARS-CoV-2 WBE, reaching a detection limit of 10 gc µL^−1^ according to the conditions surrounding the virus itself and the RNA fragments derived from it in nature.

## Figures and Tables

**Table 1:** Sensitivity performance expressed as percentage of positive samples detected over 40 cycles at four concentrations between nCoV_IP4-14095 and 2019-nCoV_N2 primer–probe sets.

		Standard Curve Points				
RT Methodology	Gene	10^4^	10^3^	10^2^	10^1^	NTC
2-step (6-mers primers)	orf1ab	10/10 (100%)	8/8 (100%)	7/9 (77.78%)	7/10 (70%)	0/10 (0%)
	3′ N	10/10 (100%)	4/10 (44.5%)	6/10 (60%)	0/10 (0%)	0/10 (0%)
1-step (specific primers)	orf1ab	9/9 (100%)	11/16 (68.7%)	6/9 (66.6%)	4/10 (40%)	1/10 (10%)
	3′ N	9/10 (90%)	8/9 (88.9%)	8/10 (80%)	6/10 (60%)	0/10 (0%)

## Data Availability

No new data were created or analyzed in this study.

## Supplementary Materials

The following supporting information can be downloaded at: https://www.mdpi.com/article/10.3390/ijms25158071/s1.

## References

[1] Morawska L., Cao J. (2020). Airborne Transmission of SARS-CoV-2: The World Should Face the Reality. Environ. Int..

[2] Yu P., Zhu J., Zhang Z., Han Y. (2020). A Familial Cluster of Infection Associated with the 2019 Novel Coronavirus Indicating Possible Person-to-Person Transmission during the Incubation Period. J. Infec. Dis..

[3] Brönimann S., Rebhan K., Lemberger U., Misrai V., Shariat S.F., Pradere B. (2020). Secretion of Severe Acute Respiratory Syndrome Coronavirus 2 in Urine. Curr. Opin. Urol..

[4] Zhang J., Wang S., Xue Y. (2020). Fecal Specimen Diagnosis 2019 Novel Coronavirus–Infected Pneumonia. J. Med. Virol..

[5] Chen Y., Chen L., Deng Q., Zhang G., Wu K., Ni L., Yang Y., Liu B., Wang W., Wei C. (2020). The Presence of SARS-CoV-2 RNA in the Feces of COVID-19 Patients. J. Med. Virol..

[6] Du W., Yu J., Liu X., Chen H., Lin L., Li Q. (2020). Persistence of SARS-CoV-2 Virus RNA in Feces: A Case Series of Children. J. Infect. Public Health.

[7] Tang A., Tong Z., Wang H., Dai Y., Li K., Liu J., Wu W., Yuan C., Yu M., Li P. (2020). Detection of Novel Coronavirus by RT-PCR in Stool Specimen from Asymptomatic Child, China. Emerg. Infect. Dis..

[8] Wölfel R., Corman V.M., Guggemos W., Seilmaier M., Zange S., Müller M.A., Niemeyer D., Jones T.C., Vollmar P., Rothe C. (2020). Virological Assessment of Hospitalized Patients with COVID-2019. Nature.

[9] Xiao F., Sun J., Xu Y., Li F., Huang X., Li H., Zhao J., Huang J., Zhao J. (2020). Infectious SARS-CoV-2 in Feces of Patient with Severe COVID-19. Emerg. Infect. Dis..

[10] Jiang X., Luo M., Zou Z., Wang X., Chen C., Qiu J. (2020). Asymptomatic SARS-CoV-2 Infected Case with Viral Detection Positive in Stool but Negative in Nasopharyngeal Samples Lasts for 42 Days. J. Med. Virol..

[11] Collivignarelli M.C., Collivignarelli C., Carnevale Miino M., Abbà A., Pedrazzani R., Bertanza G. (2020). SARS-CoV-2 in Sewer Systems and Connected Facilities. Process Saf. Environ. Prot..

[12] Kitajima M., Ahmed W., Bibby K., Carducci A., Gerba C.P., Hamilton K.A., Haramoto E., Rose J.B. (2020). SARS-CoV-2 in Wastewater: State of the Knowledge and Research Needs. Sci. Total Environ..

[13] Ahmed W., Angel N., Edson J., Bibby K., Bivins A., O’Brien J.W., Choi P.M., Kitajima M., Simpson S.L., Li J. (2020). First Confirmed Detection of SARS-CoV-2 in Untreated Wastewater in Australia: A Proof of Concept for the Wastewater Surveillance of COVID-19 in the Community. Sci. Total Environ..

[14] Asghar H., Diop O.M., Weldegebriel G., Malik F., Shetty S., El Bassioni L., Akande A.O., Al Maamoun E., Zaidi S., Adeniji A.J. (2014). Environmental Surveillance for Polioviruses in the Global Polio Eradication Initiative. J. Infect. Dis..

[15] Hellmér M., Paxéus N., Magnius L., Enache L., Arnholm B., Johansson A., Bergström T., Norder H. (2014). Detection of Pathogenic Viruses in Sewage Provided Early Warnings of Hepatitis A Virus and Norovirus Outbreaks. Appl. Environ. Microbiol..

[16] Wigginton K.R., Ye Y., Ellenberg R.M. (2015). Emerging Investigators Series: The Source and Fate of Pandemic Viruses in the Urban Water Cycle. Environ. Sci. Water Res. Technol..

[17] Barceló D. (2020). An Environmental and Health Perspective for COVID-19 Outbreak: Meteorology and Air Quality Influence, Sewage Epidemiology Indicator, Hospitals Disinfection, Drug Therapies and Recommendations. J. Environ. Chem. Eng..

[18] Randazzo W., Truchado P., Cuevas-Ferrando E., Simón P., Allende A., Sánchez G. (2020). SARS-CoV-2 RNA in Wastewater Anticipated COVID-19 Occurrence in a Low Prevalence Area. Water Res..

[19] Medema G., Heijnen L., Elsinga G., Italiaander R., Brouwer A. (2020). Presence of SARS-Coronavirus-2 RNA in Sewage and Correlation with Reported COVID-19 Prevalence in the Early Stage of the Epidemic in the Netherlands. Environ. Sci. Technol. Lett..

[20] Lodder W., de Roda Husman A.M. (2020). SARS-CoV-2 in Wastewater: Potential Health Risk, but Also Data Source. Lancet Gastroenterol. Hepatol..

[21] Wurtzer S., Marechal V., Mouchel J.M., Maday Y., Teyssou R., Richard E., Almayrac J.L., Moulin L. (2020). Evaluation of Lockdown Effect on SARS-CoV-2 Dynamics through Viral Genome Quantification in Waste Water, Greater Paris, France, 5 March to 23 April 2020. Eurosurveillance.

[22] Haramoto E., Malla B., Thakali O., Kitajima M. (2020). First Environmental Surveillance for the Presence of SARS-CoV-2 RNA in Wastewater and River Water in Japan. Sci. Total Environ..

[23] Kumar M., Patel A.K., Shah A.V., Raval J., Rajpara N., Joshi M., Joshi C.G. (2020). First Proof of the Capability of Wastewater Surveillance for COVID-19 in India through Detection of Genetic Material of SARS-CoV-2. Sci. Total Environ..

[24] La Rosa G., Iaconelli M., Mancini P., Bonanno Ferraro G., Veneri C., Bonadonna L., Lucentini L., Suffredini E. (2020). First Detection of SARS-CoV-2 in Untreated Wastewaters in Italy. Sci. Total Environ..

[25] Jafferali M.H., Khatami K., Atasoy M., Birgersson M., Williams C., Cetecioglu Z. (2021). Benchmarking Virus Concentration Methods for Quantification of SARS-CoV-2 in Raw Wastewater. Sci. Total Environ..

[26] Sherchan S.P., Shahin S., Ward L.M., Tandukar S., Aw T.G., Schmitz B., Ahmed W., Kitajima M. (2020). First Detection of SARS-CoV-2 RNA in Wastewater in North America: A Study in Louisiana, USA. Sci. Total Environ..

[27] Chu D.T., Singh V., Vu Ngoc S.M., Nguyen T.L., Barceló D. (2022). Transmission of SARS-CoV-2 Infections and Exposure in Surfaces, Points and Wastewaters: A Global One Health Perspective. Case Stud. Chem. Environ. Eng..

[28] Parra-Arroyo L., Martínez-Ruiz M., Lucero S., Oyervides-Muñoz M.A., Wilkinson M., Melchor-Martínez E.M., Araújo R.G., Coronado-Apodaca K.G., Velasco Bedran H., Buitrón G. (2023). Degradation of Viral RNA in Wastewater Complex Matrix Models and Other Standards for Wastewater-Based Epidemiology: A Review. Trends Analyt Chem..

[29] Quilliam R.S., Weidmann M., Moresco V., Purshouse H., O’Hara Z., Oliver D.M. (2020). COVID-19: The Environmental Implications of Shedding SARS-CoV-2 in Human Faeces. Environ. Int..

[30] Termansen M.B., Frische S. (2023). Fecal-Oral Transmission of SARS-CoV-2: A Systematic Review of Evidence from Epidemiological and Experimental Studies. Am. J. Infect. Control.

[31] Maal-Bared R., Brisolara K., Knight M., Mansfeldt C. (2023). To Sample or Not to Sample: A Governance-Focused Decision Tree for Wastewater Service Providers Considering Participation in Wastewater-Based Epidemiology (WBE) in Support of Public Health Programs. Sci. Total Environ..

[32] Ahmed W., Simpson S.L., Bertsch P.M., Bibby K., Bivins A., Blackall L.L., Bofill-Mas S., Bosch A., Brandão J., Choi P.M. (2022). Minimizing Errors in RT-PCR Detection and Quantification of SARS-CoV-2 RNA for Wastewater Surveillance. Sci. Total Environ..

[33] Tiwari A., Radu E., Kreuzinger N., Ahmed W., Pitkänen T. (2024). Key Considerations for Pathogen Surveillance in Wastewater. Sci. Total Environ..

[34] Uddin M., Mustafa F., Rizvi T.A., Loney T., Al Suwaidi H., Al-Marzouqi A.H.H., Eldin A.K., Alsabeeha N., Adrian T.E., Stefanini C. (2020). SARS-CoV-2/COVID-19: Viral Genomics, Epidemiology, Vaccines, and Therapeutic Interventions. Viruses.

[35] Bustin S., Nolan T. (2017). Talking the Talk, but Not Walking the Walk: RT-qPCR as a Paradigm for the Lack of Reproducibility in Molecular Research. Eur. J. Clin. Investig..

[36] Singer A.C., Thompson J.R., Filho C.R.M., Street R., Li X., Castiglioni S., Thomas K.V. (2023). A World of Wastewater-Based Epidemiology. Nat. Water.

[37] Tiwari A., Adhikari S., Zhang S., Solomon T.B., Lipponen A., Islam M.A., Thakali O., Sangkham S., Shaheen M.N.F., Jiang G. (2023). Tracing COVID-19 Trails in Wastewater: A Systematic Review of SARS-CoV-2 Surveillance with Viral Variants. Water.

[38] Parkins M.D., Lee B.E., Acosta N., Bautista M., Hubert C.R.J., Hrudey S.E., Frankowski K., Pang X.-L. (2023). Wastewater-Based Surveillance as a Tool for Public Health Action: SARS-CoV-2 and Beyond. Clin. Microbiol. Rev..

[39] Peccia J., Zulli A., Brackney D.E., Grubaugh N.D., Kaplan E.H., Casanovas-Massana A., Ko A.I., Malik A.A., Wang D., Wang M. (2020). Measurement of SARS-CoV-2 RNA in Wastewater Tracks Community Infection Dynamics. Nat. Biotechnol..

[40] Odonkor S.T., Ampofo J.K. (2013). Escherichia Coli as an Indicator of Bacteriological Quality of Water: An Overview. Microbiol. Res..

[41] Zhan Q., Babler K.M., Sharkey M.E., Amirali A., Beaver C.C., Boone M.M., Comerford S., Cooper D., Cortizas E.M., Currall B.B. (2022). Relationships between SARS-CoV-2 in Wastewater and COVID-19 Clinical Cases and Hospitalizations, with and without Normalization Against Indicators of Human Waste. ACS ES&T-Water.

[42] Hjelmsø M.H., Hellmér M., Fernandez-Cassi X., Timoneda N., Lukjancenko O., Seidel M., Elsässer D., Aarestrup F.M., Löfström C., Bofill-Mas S. (2017). Evaluation of Methods for the Concentration and Extraction of Viruses from Sewage in the Context of Metagenomic Sequencing. PLoS ONE.

[43] Ribeiro A.V.C., Mannarino C.F., de Castro E.S.G., Prado T., Ferreira F.C., Fumian T.M., Miagostovich M.P. (2023). Assessment of Virus Concentration Methods for Detecting SARS-CoV-2 in Wastewater. Braz. J. Microbiol..

[44] John S.G., Mendez C.B., Deng L., Poulos B., Kauffman A.K.M., Kern S., Brum J., Polz M.F., Boyle E.A., Sullivan M.B. (2011). A Simple and Efficient Method for Concentration of Ocean Viruses by Chemical Flocculation. Environ. Microbiol. Rep..

[45] Ciannella S., González-Fernández C., Gomez-Pastora J. (2023). Recent Progress on Wastewater-Based Epidemiology for COVID-19 Surveillance: A Systematic Review of Analytical Procedures and Epidemiological Modeling. Sci. Total Environ..

[46] Barcellos D.S., Barquilha C.E.R., Oliveira P.E., Prokopiuk M., Etchepare R.G. (2023). How Has the COVID-19 Pandemic Impacted Wastewater-Based Epidemiology?. Sci. Total Environ..

[47] Gogoi G., Singh S.D., Kalyan E., Koch D., Gogoi P., Kshattry M., Mahanta H.J., Imran M., Pandey R., Bharali P. (2024). An Interpretative Review of the Wastewater-Based Surveillance of the SARS-CoV-2: Where Do We Stand on Its Presence and Concern?. Front. Microbiol..

[48] Westhaus S., Weber F.A., Schiwy S., Linnemann V., Brinkmann M., Widera M., Greve C., Janke A., Hollert H., Wintgens T. (2021). Detection of SARS-CoV-2 in Raw and Treated Wastewater in Germany—Suitability for COVID-19 Surveillance and Potential Transmission Risks. Sci. Total Environ..

[49] Alygizakis N., Markou A.N., Rousis N.I., Galani A., Avgeris M., Adamopoulos P.G., Scorilas A., Lianidou E.S., Paraskevis D., Tsiodras S. (2021). Analytical Methodologies for the Detection of SARS-CoV-2 in Wastewater: Protocols and Future Perspectives. TrAC Trends Anal. Chem..

[50] Crits-Christoph A., Kantor R.S., Olm M.R., Whitney O.N., Al-Shayeb B., Lou Y.C., Flamholz A., Kennedy L.C., Greenwald H., Hinkle A. (2021). Genome Sequencing of Sewage Detects Regionally Prevalent SARS-CoV-2 Variants. mBio.

[51] Izquierdo-Lara R., Elsinga G., Heijnen L., Munnink B.B.O., Schapendonk C.M.E., Nieuwenhuijse D., Kon M., Lu L., Aarestrup F.M., Lycett S. (2021). Monitoring SARS-CoV-2 Circulation and Diversity through Community Wastewater Sequencing, the Netherlands and Belgium. Emerg. Infect. Dis..

[52] Jahn K., Dreifuss D., Topolsky I., Kull A., Ganesanandamoorthy P., Fernandez-Cassi X., Bänziger C., Devaux A.J., Stachler E., Caduff L. (2022). Early Detection and Surveillance of SARS-CoV-2 Genomic Variants in Wastewater Using COJAC. Nat. Microbiol..

[53] Martin J., Klapsa D., Wilton T., Zambon M., Bentley E., Bujaki E., Fritzsche M., Mate R., Majumdar M. (2020). Tracking SARS-CoV-2 in Sewage: Evidence of Changes in Virus Variant Predominance during COVID-19 Pandemic. Viruses.

[54] Gonzalez R., Curtis K., Bivins A., Bibby K., Weir M.H., Yetka K., Thompson H., Keeling D., Mitchell J., Gonzalez D. (2020). COVID-19 Surveillance in Southeastern Virginia Using Wastewater-Based Epidemiology. Water Res..

[55] Amoah I.D., Mthethwa N.P., Pillay L., Deepnarain N., Pillay K., Awolusi O.O., Kumari S., Bux F. (2021). RT-LAMP: A Cheaper, Simpler and Faster Alternative for the Detection of SARS-CoV-2 in Wastewater. Food Environ. Virol..

[56] Bivins A., Lott M., Shaffer M., Wu Z., North D., Lipp E.K., Bibby K. (2021). Building-Level Wastewater Surveillance Using Tampon Swabs and RT-LAMP for Rapid SARS-CoV-2 RNA Detection. Environ. Sci. Water Res. Technol..

[57] Francois P., Tangomo M., Hibbs J., Bonetti E.J., Boehme C.C., Notomi T., Perkins M.D., Schrenzel J. (2011). Robustness of a Loop-Mediated Isothermal Amplification Reaction for Diagnostic Applications. FEMS Immunol. Med. Microbiol..

[58] La Rosa G., Mancini P., Bonanno Ferraro G., Veneri C., Iaconelli M., Bonadonna L., Lucentini L., Suffredini E. (2021). SARS-CoV-2 Has Been Circulating in Northern Italy since December 2019: Evidence from Environmental Monitoring. Sci. Total Environ..

[59] Rimoldi S.G., Stefani F., Gigantiello A., Polesello S., Comandatore F., Mileto D., Maresca M., Longobardi C., Mancon A., Romeri F. (2020). Presence and Infectivity of SARS-CoV-2 Virus in Wastewaters and Rivers. Sci. Total Environ..

[60] Alves P.A., de Oliveira E.G., Franco-Luiz A.P.M., Almeida L.T., Gonçalves A.B., Borges I.A., Rocha F.d.S., Rocha R.P., Bezerra M.F., Miranda P. (2021). Optimization and Clinical Validation of Colorimetric Reverse Transcription Loop-Mediated Isothermal Amplification, a Fast, Highly Sensitive and Specific COVID-19 Molecular Diagnostic Tool That Is Robust to Detect SARS-CoV-2 Variants of Concern. Front. Microbiol..

[61] Bivins A., Kaya D., Bibby K., Simpson S.L., Bustin S.A., Shanks O.C., Ahmed W. (2021). Variability in RT-qPCR Assay Parameters Indicates Unreliable SARS-CoV-2 RNA Quantification for Wastewater Surveillance. Water Res..

[62] Zhang S., Li X., Shi J., Sivakumar M., Luby S., O’Brien J., Jiang G. (2022). Analytical Performance Comparison of Four SARS-CoV-2 RT-qPCR Primer-Probe Sets for Wastewater Samples. Sci. Total Environ..

[63] Li X., Zhang S., Shi J., Luby S.P., Jiang G. (2021). Uncertainties in Estimating SARS-CoV-2 Prevalence by Wastewater-Based Epidemiology. Chem. Eng. J..

[64] Van Dusen J., LeBlanc H., Nastasi N., Panescu J., Shamblin A., Smith J.W., Sovic M.G., Williams A., Quam M.B.M., Faith S. (2024). Identification of SARS-CoV-2 Variants in Indoor Dust. PLoS ONE.

[65] Huisman J.S., Scire J., Caduff L., Fernandez-Cassi X., Ganesanandamoorthy P., Kull A., Scheidegger A., Stachler E., Boehm A.B., Hughes B. (2022). Wastewater-Based Estimation of the Effective Reproductive Number of SARS-CoV-2. Environ. Health Perspect..

[66] Lipps W.C., Braun-Howland E.B., Baxter T.E., American Public Health Association, American Water Works Association, Water Environment Federation (2023). Standard Methods for the Examination of Water and Wastewater.

[67] Johns Hopkins CSSE COVID-19 Dashboard by the Center for Systems Science and Engineering (CSSE) at Johns Hopkins University (JHU). ArcGIS. Johns Hopkins University. https://gisanddata.maps.arcgis.com/apps/opsdashboard/index.html#/bda7594740fd40299423467b48e9ecf6.

[68] García-Bernalt Diego J., Fernández-Soto P., Domínguez-Gil M., Belhassen-García M., Bellido J.L.M., Muro A. (2021). A Simple, Affordable, Rapid, Stabilized, Colorimetric, Versatile RT-LAMP Assay to Detect SARS-CoV-2. Diagnostics.

